# The Plant Short-Chain Dehydrogenase (SDR) superfamily: genome-wide inventory and diversification patterns

**DOI:** 10.1186/1471-2229-12-219

**Published:** 2012-11-20

**Authors:** Hanane Moummou, Yvonne Kallberg, Libert Brice Tonfack, Bengt Persson, Benoît van der Rest

**Affiliations:** 1 Université de Toulouse, INPT-ENSAT, UMR990 Génomique et Biotechnologie des Fruits, Avenue de l’Agrobiopole, BP 32607, Castanet-Tolosan, F-31326, France; 2 Laboratory of Food Science, Faculty of Science Semlalia, University CADI AYYAD, Marrakech, Morocco; 3 Bioinformatics Infrastructure for Life Sciences, Science for Life Laboratory, Centre for Molecular Medicine, Karolinska Institutet, SE-171 77, Stockholm, Sweden; 4 Laboratory of Biotechnology and Environment, Unit of Plant Physiology and Improvement, Department of Plant Biology, Faculty of Science, University of Yaounde 1, PO BOX 812, Yaounde, Cameroon; 5 Science for Life Laboratory, Department of Cell and Molecular Biology (CMB), Karolinska Institutet, SE-17177, Stockholm, Sweden; 6 IFM Bioinformatics and Swedish e-Science Research Centre (SeRC), Linköping University, SE-58183, Linköping, Sweden; 7 INRA, UMR990 Génomique et Biotechnologie des Fruits, 24 Chemin de Borde Rouge, Castanet-Tolosan, F-31326, France

**Keywords:** Short-chain dehydrogenase/reductase (SDRs), SDR nomenclature initiative, Hidden markov model, Multigenic family, Plant

## Abstract

**Background:**

Short-chain dehydrogenases/reductases (SDRs) form one of the largest and oldest NAD(P)(H) dependent oxidoreductase families. Despite a conserved ‘Rossmann-fold’ structure, members of the SDR superfamily exhibit low sequence similarities, which constituted a bottleneck in terms of identification. Recent classification methods, relying on hidden-Markov models (HMMs), improved identification and enabled the construction of a nomenclature. However, functional annotations of plant SDRs remain scarce.

**Results:**

Wide-scale analyses were performed on ten plant genomes. The combination of hidden Markov model (HMM) based analyses and similarity searches led to the construction of an exhaustive inventory of plant SDR. With 68 to 315 members found in each analysed genome, the inventory confirmed the over-representation of SDRs in plants compared to animals, fungi and prokaryotes. The plant SDRs were first classified into three major types — ‘classical’, ‘extended’ and ‘divergent’ — but a minority (10% of the predicted SDRs) could not be classified into these general types (‘unknown’ or ‘atypical’ types). In a second step, we could categorize the vast majority of land plant SDRs into a set of 49 families. Out of these 49 families, 35 appeared early during evolution since they are commonly found through all the Green Lineage. Yet, some SDR families — tropinone reductase-like proteins (SDR65C), ‘ABA2-like’-NAD dehydrogenase (SDR110C), ‘salutaridine/menthone-reductase-like’ proteins (SDR114C), ‘dihydroflavonol 4-reductase’-like proteins (SDR108E) and ‘isoflavone-reductase-like’ (SDR460A) proteins — have undergone significant functional diversification within vascular plants since they diverged from Bryophytes. Interestingly, these diversified families are either involved in the secondary metabolism routes (terpenoids, alkaloids, phenolics) or participate in developmental processes (hormone biosynthesis or catabolism, flower development), in opposition to SDR families involved in primary metabolism which are poorly diversified.

**Conclusion:**

The application of HMMs to plant genomes enabled us to identify 49 families that encompass all Angiosperms (‘higher plants’) SDRs, each family being sufficiently conserved to enable simpler analyses based only on overall sequence similarity. The multiplicity of SDRs in plant kingdom is mainly explained by the diversification of large families involved in different secondary metabolism pathways, suggesting that the chemical diversification that accompanied the emergence of vascular plants acted as a driving force for SDR evolution.

## Background

Short-chain dehydrogenases/reductases (SDRs) constitute one of the largest and oldest protein superfamilies known to date. This ancient family, found in all domains of life (Archea, Eukaryotes, Prokaryotes and viruses), is characterized by large sequence divergences but several common properties: (i) a conserved 3D structure consisting of ‘Rossmann-fold’ β-sheet with α-helices on both sides, (ii) an N-terminal dinucleotide cofactor binding motif, (iii) an active site with a catalytical residue motif YxxxK [1,2]. With the release of genome sequences of numerous living organisms, the availability of around 300 crystal structures and the identification of many enzymatic functions, much attention has been given to classify the members of the SDR superfamily. A first discrimination was established between five types of SDR: the ‘classical’ type, consisting of approximately 250 amino acids, the ‘extended’ type that has an additional 100-residue domain in the C-terminal region, the ‘intermediate’ type that displays a specific G/AxxGxxG/A cofactor binding motif, the ‘divergent’ type that comprises enoyl-reductases from plant and bacteria and harbours modifications both in the cofactor binding site and active site motifs and the ‘complex’ SDR which are usually part of large multi-domain enzymes, such as mammalian fatty acid synthases or bacterial polyketide synthases [2-4]. Moreover, the discovery of new oxidoreductase structures harbouring the SDR ‘Rossmann-fold’ motif revealed the existence of uncommon types, often referred to as ‘unknown’ or ‘atypical’ types. More recently, the diversity of SDRs, either their amino acid sequences or their functions, led to the development of a second classification effort: the ‘SDR Nomenclature Initiative’ that aims at being more informative regarding SDRs functions and at establishing a sustainable and expandable nomenclature system based on the use of a large set of hidden Markov models (HMM) [5]. Nowadays, 449 families have been listed in this nomenclature [6].

Although mentioned by several authors [2,4], the diversity of SDRs in plants has never been investigated thoroughly. The recent advances in sequencing techniques and the still-increasing speed of genome releases now facilitate an exhaustive review of complex multigenic families. In the case of SDRs, a second challenge for plant scientific community is to unravel the functions of these oxidoreductases. Indeed, in the TAIR10 annotation of *Arabidopsis thaliana* genome, a large majority of ‘classical’ SDRs (two thirds) are merely annotated as NAD(P)-binding Rossmann-fold superfamily protein oxidoreductase [7]. This lack of information prompted us to adopt an exhaustive approach on plant SDRs. In a previous paper, we reviewed the involvement of different SDRs in primary and secondary metabolism [8]. In the present paper, we combined the use of HMMs and phylogenetic analyses on a set of genomes representative of plant diversity, in order to conduct a global inventory of plant SDRs coherent with the current SDR classification and nomenclature. This inventory was integrated into a functional classification of plant SDRs. Since this genome-wide inventory confirmed the high diversity of plant SDRs, the distribution and evolution of the different SDR families was examined, notably to investigate the link between SDR diversification and the emergence of secondary metabolism in vascular plants.

## Methods

### Analysed genomes

Genome analyses were performed on ten distinct genomes comprising four Dicots, three Monocots, the Pteridophytae *Selagniella moellendorffii*, the moss *Physcomitrella patens* and the Alga *Chlamydomonas reinhardtii*. Sequences and most annotations were downloaded from the Joint Genome Institute website. The predicted proteomes analysed [7,9-17] were deduced from the annotations given in Table 1.

**Table 1:** Reference and size of the analyzed genomes

**Species**	**Taxa**	**Annotation used**	**Number of loci**	**Reference**
*Chlamydomonas reinhardtii*	Chlorophyte	Chlre4.1_Augustus9	15935	[9]
*Physcomitrella patens*	Moss	proteins. Phypa1_1.FilteredModels	35938	[10]
*Selaginella moellendorffii*	Lycophyte	Selmo1_GeneModels_FilteredModels3	22285	[11]
*Arabidopsis thaliana*	Eudicot	TAIR9	27379	[7]
*Populus trichocarpo*	Eudicot	Populus.trichocarpa.v2.0	41377	[12]
*Vitis vinifera*	Eudicot	12X March 2010 release	26346	[13]
*Glycine max*	Eudicot	Glyma1_pacId	46367	[14]
*Oryza sativa*	Monocot	MSU Rice Genome Annotation (Osa1) Release 6.1	40577	[15]
*Zea mays*	Monocot	ZmB73_4a.53_working_translations	102202	[16]
*Sorghum bicolor*	Monocot	Sorbi1_GeneModels_Sbi1_4_aa	34496	[17]

The number of loci corresponds to the protein coding genes predicted by the annotation.

### HMM-based analyses of plant genomes

Genomic sets of predicted proteins were challenged with three Pfams HMMs [18]: PF00106, PF01370 and PF01073 using HMMER3. SDR Nomenclature Initiative HMMs were defined and updated as described previously [5]. The five SDR types (‘classical’, ‘extended’, ‘intermediate’, ‘divergent’, and ‘complex’) each has an HMM trained to identify sequences of respective type. The HMMs were created using HMMER3, with manually adjusted alignments of representative sequences as seed. Cutoffs are used to decide if a hit is significant or not: ‘classical’ — 138, ‘extended’ — 108, ‘intermediate’ — 162, ‘divergent’ — 160, and ‘complex’ — 140. In addition to the five types, an ‘unknown’ label is used for sequences with scores lower that these cutoffs but still high enough to safely predict the sequence as an SDR: ‘classical’ — 29, ‘extended’ — 75, and ‘divergent’ — 100. Scores below the cutoffs are considered not positive.

For the PLR/IFR family, an HMM was created and incorporated to the ‘SDR Nomenclature Initiative’ set of family HMMs. The procedure for training the HMM was the same as previously developed with iterative refinement of the model until no new members were found [5].

### Decision rules for SDR inventory

**Figure 1:**
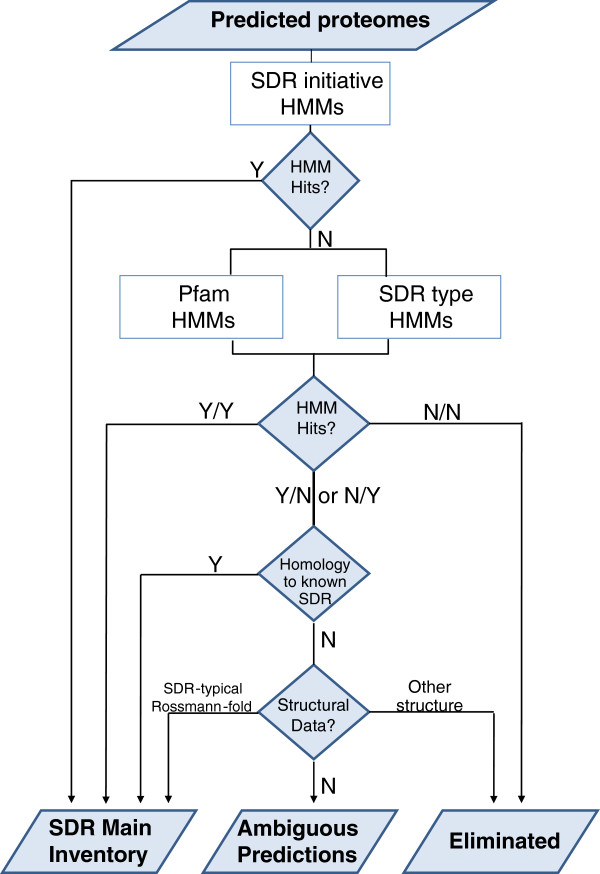
**Decision rules used to make an inventory of plant SDRs using three sets of HMM.** All the HMM sets were run independently on the 10 predicted proteomes. The complete inventory and the ambiguous predictions are included as supplementary material (Additional file 2: Table S2 and Additional file 1: Table S1).

For each sequence recognized by a HMM (hit), a score was assigned. Yet, several sequences were only recognized by one or two HMMs (either the Pfam derived HMM or the SDR-type HMM) and sometimes with a very low score. Thus, we defined a series of rules schematized in a decision tree (Figure 1). Sequences that were recognized by SDR nomenclature initiative were directly considered as positive. Sequences identified with both remaining sets of HMMs were also considered as positive. For the remaining sequences recognized only by one HMM, we first looked at the existence of strong homology with positive hits identified in the previous steps in order to include putative ‘truncated’ proteins (see Inventory refinement, below). Alternatively, we checked individually the existence of structural data in the scientific literature, which allowed either including some hits in our inventory or discarding certain families of enzymes, notably the medium-chain dehydrogenases, that display distinct structural motifs. In absence of structural data, the sequences recognized by a single HMM could not be classified and were included in a list of ambiguous sequences (Additional file 1: Table S1) that contains oxido-reductases that still await structural data before confirming or infirming their belonging to the SDR superfamily.

### Inventory refinement

For the gene loci that are associated with several gene models and therefore with different protein predictions, a sole amino acid sequence was selected according two criteria: (1) the maximum HMM score and (2) the maximum alignment score deduced from a BlastP performed on other plant genomes. When the HMM and BlastP analyses led to contradictory predictions, a single protein prediction was manually selected after aligning the different gene models with its closest homologues. To include in the SDR classification the truncated proteins that failed to be recognized by the HMMs, a BlastP sequence search was performed on each genome using as query sequences the complete list of SDRs recognized in the first round of HMM searches. All sequences that displayed a segment of 60 amino acids with more than 50% identity were classified in the same type or family as its closest homologue.

### Distance matrices and phylogenetic analyses

Phylogenetic analyses and distance matrices were built using the Mega5 package [19]. Full length amino acid sequences were aligned using the ClustalW algorithm. Distance matrices evaluating the percentage of sequence identity were calculated on the basis of p-distance with the pairwise deletion option. Unless stated differently, phylogenetic trees were built using the Neighbor-Joining method. The percentage of replicate trees in which the associated taxa clustered together was calculated in the bootstrap test (500 replicates). Trees were drawn to scale, with branch lengths in the same units as those of the evolutionary distances used to infer the phylogenetic tree. The evolutionary distances were computed using the Poisson correction method and were expressed as the units of the number of amino acid substitutions per site.

### Statistical analyses

**Figure 2:**
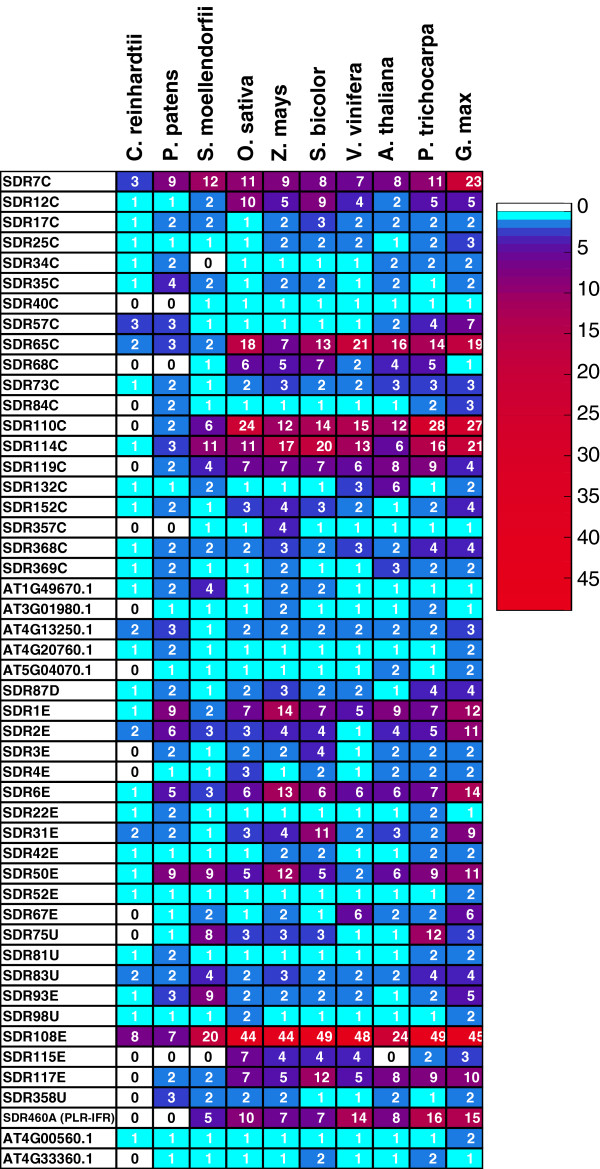
**Distribution of the SDR families in the analyzed plant genomes represented as a heat map.** The heat map was built on a distribution matrix deduced from the inventory classification shown in Additional file 2: Table S2. The blue to red color gradient reflects the number of SDR listed in each family in the different genomes; the absence of family is indicated with a white square. The names of the families were deduced from the ‘SDR Nomenclature Initiative’ HMMs or by a representative gene accession for orphan families not recognized by a specific HMM.

Principal Component Analysis was performed on the distribution matrix given in Figure 2 using the R 2.14.1 software [20] with in-house developed scripts (Elie Maza, personnal communication). The robustness of the conclusions was checked by carrying the same analysis after removal of the individual exhibiting extreme values (SDR108E).

## Results and discussion

### HMM-driven inventory of plant SDR

Initial HMM analyses were performed on ten complete genomes: 4 Eudicots (*Arabidopsis thaliana*, *Populus trichocarpa*, *Vitis vinifera*, *Glycine max*), 3 Monocots (*Zea mays*, *Oryza sativa* and *Sorghum bicolor*), the lycophyte *Selaginella moellendorffii*, the moss *Physcomitrella patens* and the unicellular green alga *Chlamydomonas reinhardtii* (Table 1). The predicted ‘proteomes’ deduced from the genome annotations were searched against three distinct sets of HMMs: the Pfam HMMs considered to encompass most SDR (PF00106, PF01370, PF01073), HMMs developed in the framework of the SDR nomenclature initiative [5,21] and a set of HMMs developed to predict the type (‘classical’, ‘extended’, ‘intermediary’, ‘divergent’ and ‘complex’) of SDR (see Methods).

This first analysis led to an exhaustive inventory of plant SDRs presented in supplemental data (Additional file 1: Table S1 and Additional file 2: Table S2). This inventory was divided into a main list (Additional file 1: Table S1), where the HMM scores or the high similarity with known SDRs were sufficient to establish a good prediction, and a complementary list of ambiguous SDR predictions (Additional file 2: Table S2), containing proteins with low HMM scores and absence of structural data (see decision tree in Figure 1 and Methods). Despite its very low HMM scores, we included in the main list a large family that comprises pinoresinol reductase (PLR), isoflavone reductase (IFR), vestitone reductase, phenylcoumaran benzylic ether reductase and eugenol synthase. Indeed, the structures of several members of this family were resolved by crystallography and the data revealed the presence of a SDR-typical Rossmann-fold [22-25]. Subsequently, an HMM was created and incorporated to the ‘SDR Nomenclature Initiative’ set of HMMs. The PLR/IFR family was named SDR460A, where the ‘A’ stands for ‘atypical’.

### Distribution of plant SDRs

The number and the distribution of plant SDRs of different types are summarized in Table 2. As in other Eukaryotes, the major types consist of ‘classical’ and ‘extended’ SDRs. ‘Divergent’ SDRs are limited to one conserved family: an enoyl-ACP reductase (ENR) involved in lipid biosynthesis (AT2G05990.1 in *Arabidopsis*) [8,26]. While neither ‘intermediate’ nor ‘complex’ types are found in plants, we can notice a high number of ‘unknown’ types, meaning that the sequence patterns clearly differ from the other types. As previously noticed [2], the SDR family is highly represented in the plant kingdom: while 73 SDRs were numbered in the human genome [27] and 39 in the cyanobacteria *Synechocystis* sp. PCC 6803 [28], the number of SDRs in land plants vary from 126 in the moss *P. patens* to 315 in soybean (*G. max*). Even if we consider the variations due to the genome sizes (Table 1), SDRs are more represented in the Angiosperms than in the alga *C. reinhardtii* or in *P. patens,* suggesting a relationship between the emergence of vascular plant and the apparent multiplicity of plant SDRs.

**Table 2:** Distribution of SDRs in different plants

	**Total SDR**	**Types of SDR**				
		**Classical**	**Divergent**	**Extended**	**Atypical (PLR-IFR)**	**Unknown**
**Arabidopsis**	**178**	90	1	72	8	7
**Poplar**	**268**	122	4	106	16	20
**Grapevine**	**205**	95	2	88	14	6
**Soybean**	**315**	145	4	138	15	13
**Rice**	**227**	110	2	95	10	10
**Maize**	**230**	97	3	113	7	10
**Sorghum**	**237**	106	2*	114	7	8
**Selaginella**	**142**	64	1	55	5	17
**Physcomitrella**	**126**	59	2	55	0	10
**Chlamydomonas**	**68**	41	1	21	1	4

Families with low scores and no structural data (listed in Table S2) were omitted. *The presence of divergent SDRs in *Sorghum bicolor* was deduced from the Sbicolor_79_peptide annotation.

### Sub-classification of plant SDR

The HMMs developed by the SDR nomenclature initiative [5] aim at classifying the SDR superfamily into a large number of families, at a level where this classification would be informative regarding the functions of SDRs. In a first analysis, the HMMs defined in the frame of the SDR nomenclature initiative directly recognized 74% of plant SDRs. After performing similarity searches and associating truncated proteins with its closest homologues (see Methods), the proportion of non-classified SDRs dropped markedly since 94.5% of plant SDRs were categorized into 49 families. While the majority of these families are found in most Tracheophytes (Table 3), seven (SDR58C, 59C, 74C, 86C, 90C, 103U, 107C; Additional file 2: Table S2) are found only in *P. patens* or in *C. reinhardtii*. The occurrence of different family sequences in the analysed genomes was represented as a heat map (Figure 2).

**Table 3:** Classification of plant SDRs

**Representative gene**	**SDR nomenclature initiative**	**Known functions**	**Occurence**	**Average identity (%)**
AT4G23420	SDR7C	*Pisum sativum* Tic32 (chloroplast protein import translocon)	**ViridP**	49,4
AT1G67730	SDR12C	β-ketoacyl reductase (fatty acids elongation)	**LandP**	48,4
AT3G12800	SDR17C	-	**ViridP**	64,1
AT4G05530	SDR25C	SDRA-IBR1 (indole-3-butyric acid response 1)	**ViridP**	67,7
AT3G03330	SDR34C	-	**ViridP**	56,1
AT3G06060	SDR35C	-	**ViridP**	47,9
AT4G09750	SDR40C	-	ViridP*	70,8
AT1G54870	SDR57C	-	**ViridP**	58,0
AT5G06060	SDR65C	Tropinone Reductase	**ViridP**	53,3
AT3G03980	SDR68C		**TracheoP**	57,0
AT5G54190	SDR73C	Protochlorophyllide Oxidoreductase	**ViridP**	74,5
AT3G50560	SDR84C	-	**ViridP**	60,4
AT1G52340	SDR110C	ABA2 (xanthoxin oxidase), Tasselseed2, Secoisolariciresinol dehydrogenase, Momilactone A synthase, Isopiperitenol dehydrogenase	**LandP**	47,1
AT3G61220	SDR114C	*S*alutaridine reductase, Menthone reductase, Isopiperitenone reductase	**ViridP**	45,4
AT5G50600	SDR119C	Hydroxysteroid Dehydrogenase	**LandP**	44,4
AT3G55290	SDR132C	*Solanum tuberosum* TDF511	**ViridP**	62,4
AT1G24360	SDR152C	FAS-II- β-ketoacyl reductase (FabG)	**ViridP**	68,3
AT1G10310	SDR357C	Pterin aldehyde reductase (folate salvage)	**TracheoP**	70,0
AT5G10050	SDR368C	-	**ViridP**	45,8
AT4G27760	SDR369C	*Arabidopsis thaliana* Forever Young	**ViridP**	57,2
AT2G05990	SDR87D	Enoyl-ACP reductase (ENR)	**ViridP**	75,0
AT1G49670	-	-	**ViridP**	50,6
AT3G01980	-	*Cucumis melo* ADH2	**LandP**	57,8
AT4G13250	-	NYC1/NOL (chlorophyll b reductase)	**ViridP**	48,1
AT4G20760	-	-	**ViridP**	61,8
AT5G04070	-	-	**LandP**	52,7
AT4G10960	SDR1E	UDP-D-glucose/UDP-D-galactose 4-epimerase, UDP-arabinose 4-epimerase	**Virid**	55,4
AT1G78570	SDR2E	NDP-L-rhamnose synthase/epimerase	**ViridP**	74,7
AT5G66280	SDR3E	GDP-mannose 4,6-dehydratase	**LandP**	72,3
AT1G17890	SDR4E	GDP-4-keto-6-deoxymannose-3,5-epimerase-4-reductase	**LandP**	73,1
AT2G28760	SDR6E	UDP-xylose synthase, UDP-glucuronic acid decarboxylase	**ViridP**	69,7
AT2G20360	SDR22E	-	**ViridP**	60,1
AT1G47290	SDR31E	3β-hydroxysteroid-dehydrogenase/decarboxylase	**ViridP**	48,2
AT2G33630	SDR42E	-	ViridP*	66,2
AT4G30440	SDR50E	UDP-D-glucuronate 4-epimerase	**ViridP**	61,3
AT4G33030	SDR52E	UDP-sulfoquinovose synthase	**ViridP**	73,8
AT1G08200	SDR67E	UDP-D-apiose/UDP-D-xylose synthase	**LandP**	81,9
AT5G28840	SDR93E	GDP-D-mannose 3′,5′-epimerase	**ViridP**	87,4
AT5G42800	SDR108E	Dihydroflavonol 4-reductase, Anthocyanidin reductase, Cinnamoyl-CoA reductase, Phenylacetaldehyde reductase, Eutypine reductase	**ViridP**	36,6
GRMZM2G086773	SDR115E	HC-toxin reductase	**FlowerP**	55,0
AT5G22500	SDR117E	fatty-acyl-CoA reductase	**LandP**	46,8
AT4G24220	SDR75U	VEIN PATTERNING 1 (VEP1), progesterone 5β-reductase	**LandP**	53,4
AT4G35250	SDR81U	-	**ViridP**	76,4
AT1G09340	SDR83U	Chloroplast stem-loop binding protein	**ViridP**	50,7
AT5G18660	SDR98U	3,8-divinyl protochlorophyllide a 8-vinyl reductase	**ViridP**	62,5
AT5G02240	SDR358U	-	ViridP*	68,9
AT1G32100 (PLR-IFR)	SDR460A	Pinoresinol reductase, Isoflavone reductase, Vestitone reductase, Phenylcoumaran benzylic ether reductase, Eugenol synthase	**TracheoP**	45,3
AT4G33360	-	Farnesol NAD dehydrogenase	**LandP**	63,2
AT4G00560	-		**ViridP**	56,5

Each family was associated with a representative gene and, when possible, with a specific SDR nomenclature initiative HMM. Information on the occurrence of SDRs in different genomes are reported by the taxon name (ViridP: Viridiplantae; LandP: Embryophytae; TracheoP: Tracheophytae; FlowerP: Magnioliophyta). Average pairwise identities were calculated from the sequences of plant genomes. Ambiguously predicted SDRs and families absent in flowering plants were omitted. *: occurrence in Viriplantae was deduced from the presence of homologues in other Green Algae genomes.

On the opposite, 5.5% of plant SDRs (from 4% in Angiosperms to 29% in *C. reinhardtii)* remained unclassified. The existence of these orphan SDRs lays in the conception of the ‘SDR nomenclature initiative’ HMMs. In order to achieve robust HMMs, the authors considered only families with sufficient number of representative and non-redundant sequences [5], thus excluding SDR families with too few members. To circumvent this difficulty, we examined the possibility to define new families on the sole basis of amino-acid sequence conservation. Therefore, all the unclassified SDRs from the main inventory (Additional file 1: Table S1) were associated to its closest homologues using BlastP searches and sequence alignments. Interestingly, all the unclassified sequences from Angiosperms clearly matched with at least one *Arabidopsis* SDR, the e value obtained from a BlastP against *Arabidopsis* predicted proteome never exceeding 1 e-40. Thus, seven new clusters were defined on the basis of sequence conservation, four being common to all the Viridiplantae genomes while three were found only in land plants (Figure 2 and Table 3). Within these clusters, the average pairwise sequence identities ranged from 48% to 62%. These conservation rates are consistent with the average pairwise identities observed for the families defined by a ‘SDR nomenclature initiative’-HMM, that ranges from 37% to 82% identity (Table 3). All these clusters were represented by a limited number of sequences in each genome, supporting the explanation that the lack of ‘SDR-nomenclature-initiative’-HMMs is simply the consequence of an insufficient set of sequences and that these families might be defined in the future, with the release of new sequences in the UNIPROT database. To complete the plant SDR classification, each new cluster was assigned a representative gene, based on an *Arabidopsis thaliana* identifier. While all angiosperms SDRs could be categorized in a family, defined either by a specific HMM or by primary structure conservation, 15 sequences from *C. reinhardtii,* 4 sequences from *P. patens* and one sequence from *S. moellendorffii* were too distant to other SDR sequences and remained unclassified.

By extension, the ambiguous SDR sequences were also clustered on the basis of sequence homologies, allowing the definition of nine potential families (Additional file 2: Table S2). Yet, in absence of structural data confirming the existence of typical SDR structures, these sequences were not analysed further.

In a last step, plant SDR classification was combined with functional information. Taking advantage of our previous bibliographic research [8] and of the annotations found for *Arabidopsis* (TAIR10), we completed the classification by mentioning all the known functions described in the scientific literature in Table 3. Also, to each family, a representative gene was chosen according to three criteria: (1) favour *Arabidopsis* accessions with respect to the quality of TAIR annotations and its pertinence as a model plant; (2) when possible, opt for genes that have been functionally characterised; otherwise (3), priority was given to the accession that displayed the lowest average distance with other members of its family.

### Evolution and diversification of plant SDR as a potential trait of land plant emergence?

The distribution of the different families in the different taxa was further examined to understand the evolution of the plant SDR superfamily. We first addressed the question of potential origins of the different SDR families. Out of the 49 families listed in Table 3, 32 were found both in the alga *C. reinhardtii* and in the majority of land plants, suggesting that most plant SDRs families emerged prior to land plant radiation that started -460 Myear ago, in the Ordovician period [29]. For three additional families (SDR40C, SDR42E and SDR358U), the absence of a member in *C. reinhardtii* or even in *P. patens* predicted proteomes masked the occurrence of these families in other genus of green algae (*Volvox*, *Micromonas*, *Chlorella* and *Ostreococcus*), suggesting either that some genomic sets are incomplete or that the families are ancestral but the genes might have been lost in some taxa. In addition, 10 families absent in green algae are common to all land plants (Figure 2 and Table 3), indicating that 45 families are shared among land plants (embryophytes). 48 families are common to vascular plants as 3 additional families are specific to *S. moellendorffii* and Angiosperms. At last, a sole family, SDR115E, is found only in Angiosperms. The origins of some families may be very ancient: SDR1E, 2E, 6E and 7C families are found in all domains of life (Archea, Eukaryote, Prokaryote) while the SDR12C, 17C, 25C, 34C, 35C, 22E and 31E families are common to the majority of Eukaryotes [5]. Besides, several ancestral SDR families are close to Prokaryotic ‘homologues’. For example, the origin of the plastids is illustrated by the presence of chloroplastic SDRs similar to its cyanobacterial homologue. In a recent paper, Kramm *et al.*[28] listed 39 SDRs in the genome of the cyanobacteria *Synechocystis* sp. PCC 6803. 20 of these SDRs show clear homologies (>35% identity) with plant SDRs (data not shown). The SDRs clusters present both in cyanobacteria and plant genomes include the very ancient families (SDR1E, 2E, 3E, 6E) and several plastidial proteins involved in primary metabolism, such as sulfolipid biosynthesis protein (SDR52E), protochlorophyllide oxidoreductase (SDR73C), 3,8-divinyl protochlorophyllide a 8-vinyl reductase (SDR98U) or the members of the fatty acid synthase (FasII) complex (SDR152C and SDR87D).

**Figure 3:**
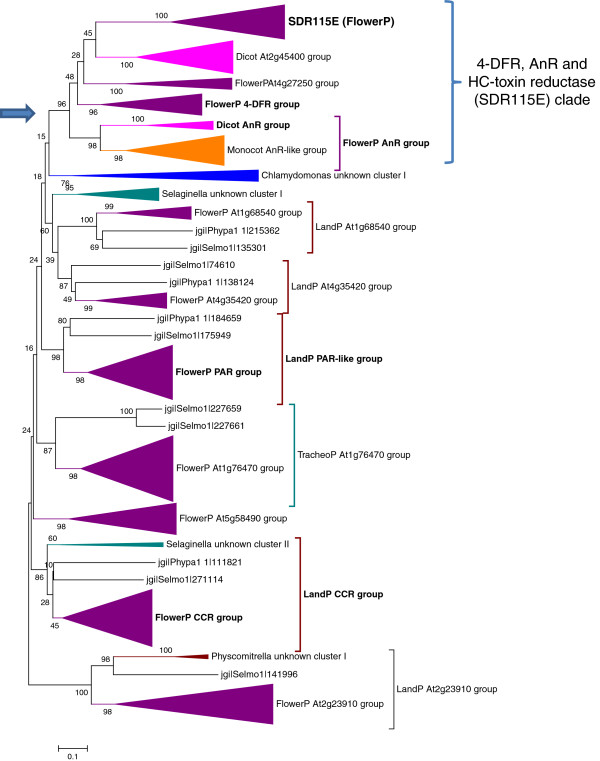
**Phylogenetic tree of the SDR108E and SDR115E families.** The blue arrow indicates the node at the origin of the ‘AnR, 4-DFR and SDR115E’ branch. Amino acid sequences recognized by the SDR108E and SDR115E HMMs were aligned with ClustalW algorithm. The evolutionary history was inferred using the Neighbor-Joining method. The percentages of replicate trees in which the associated taxa clustered together in the bootstrap test (500 replicates) are shown next to the branches. Full references of sequences compressed in different clusters are provided as supplemental data (Additional file 3: figure 1A). Consistent trees were obtained using the Maximum Likelihood method or rooting the tree with other SDR families (SDR1E, SDR6E, SDR31E) as outgroups.

The origin of these taxon-specificities probably results from three evolutionary mechanisms: horizontal gene transfers, differentiation of a novel family from a pre-existing SDR family and loss of genes. Indeed, Tarrio *et al.*[30] established that the Vein Patterning 1 (SDR75U) gene family had undergone five lateral gene transfer events, one occurring from bacteria to an ancestor of land plants. Conversely, extensive search of SDR homologues in the Genbank database revealed clear homologies between independent taxa, such as the similarities between the Tracheophyte SDR68C members and its Proteobacteria homologues or the close relationship between plant PLR-IFR family and Bacteria or Ascomycete isoflavone reductase-like proteins (data not shown), thus illustrating the possible importance of horizontal gene transfers. An original example of SDR differentiation is illustrated by the emergence of the Angiosperm-specific HC-toxin reductase (SDR115E) family, involved in the pathogen *Helminthosporium carbonum* (HC) toxin reduction [31]. Since previous phylogenetic analyses [32] showed the existence of significant homologies between HC-toxin reductase (SDR115E) and the large dihydroflavonol 4-reductase (4-DFR, SDR108E) family, we integrated SDR108E and SDR115E amino acids sequences in the same alignment and phylogenetic analysis (Figure 3 and Additional file 3: Figure S1A). The topology of the deduced tree (Figure 3 and Additional file 3: Figure S1A) suggests that the SDR115E branch belongs to a larger clade that includes 4-DFR (AT5G42800.1 cluster, [33]), anthocyanidin reductase (AT1G61720.1 cluster, [34,35]) and the brassinosteroid related 4-DFR-like protein BEN1 (AT2G45400.1 cluster, [36]). The robustness of this topology was further checked using different phylogeny algorithms (Neighbour-Joining and Maximum Likelihood) or rooting the tree with external sequences from other SDR families (SDR1E, SDR6E, SDR31E). All trees displayed similar topologies, SDR115E members always clustering with 4-DFR, anthocyanidin reductase and BEN1 (data not shown), thus supporting the view that the HC-toxin reductase (SDR115E) branch evolved from an ancestor belonging to the SDR108E family. The divergences of sequences within the SDR108E-115E ‘clade’ are sufficient to establish two distinct HMM profiles. At last, two distinct features may illustrate the loss of genes in SDR evolution: (i) although found in Monocots, grapevine, poplar and soybean genomes, the SDR115E family is absent in Arabidopsis genome or ESTs database; (ii) some families found in *P. patens* or in *S. moellendorffii* genomes (SDR74C, 86C, 103U, Additional file 2: Table S2) are absent in all the Angiosperms genomes, suggesting that genes might have been lost during before flowering plants radiation.

**Figure 4:**
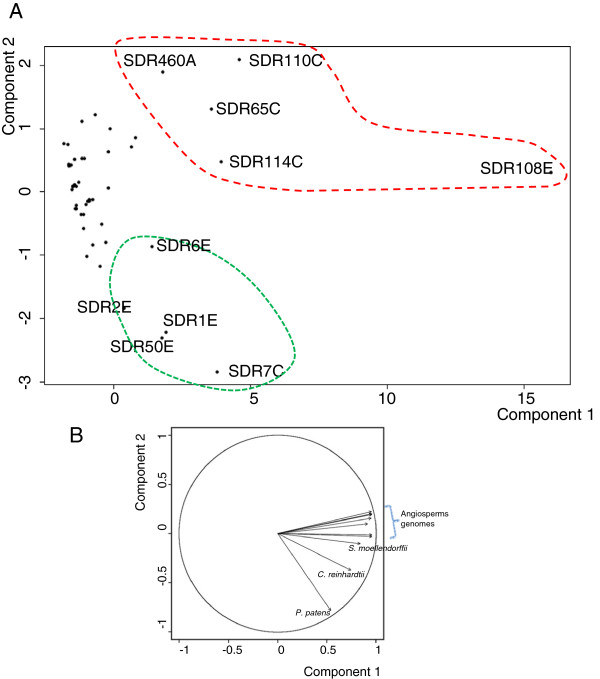
**Diversification patterns of plant SDR families deduced from Principal Component Analysis.** PCA was calculated on the distribution matrix shown in Figure 2. **A**) Scatter plot deduced from the two first components: the first and second axes respectively participate for 79% and 9% of the diversity. **B**) Contribution of different genomes (expressed as vectors) in the first and second axes values. Angiosperms genomes follow the order (anticlockwise): *G. max*, *Z. mays*, *A. thaliana*, *S. bicolor*, *P.* trichocarpa, *O. sativa*, *V. vinifera* .

The second obvious feature, when observing the distribution of SDR families (Figure 2), is the expansion pattern of the different families. A Principal Component Analysis (PCA) was performed on the distribution matrix used to build the heat map presented in Figure 2. It allowed the individualization of ten families displaying high values on the first axis (Figure 4A). All these families are characterized by a large number of members in contrast to the majority of SDR families represented in plant genomes with a limited set of sequences. Interestingly, the second axis is mainly driven by the vectors formed by *P. patens* and *C. reinhardtii* genomes (Figure 4B) and it discriminates two patterns of diversification: families expanded both in the moss *P. patens* and in vascular plants (SDR1E, SDR2E, SDR6E, SDR7C, SDR50E) and families expanded in vascular and flowering plants (SDR65C, SDR108E, SDR110C, SDR114C and SDR460A).

Remarkably, all the five families expanded in vascular plants comprise enzymes involved in secondary metabolism (Table 3): tropinone reductases (SDR65C) are known for their involvement in alkaloids biosynthesis; SDR110C NAD-dehydrogenases oxidize various phenolic or terpeninc compounds, including xanthoxin, a precursor of abscissic acid (ABA); SDR114C menthone and salutaridine reductase, are involved in monterpene and alkaloid metabolism respectively; the large SDR108E family members catalyze the reduction of several phenolic precursors (4-dihydroflavonol, anthocyanidin, cinnamoyl-CoA, phenylacetaldehyde or eutypine) and last, the atypical PLR/IFR family (SDR460A) is also involved in phenolic metabolism. On the opposite, several poorly diversified clusters (SDR52E, 73C, 152C, 87D, 357C) that contain highly conserved sequences participate in primary metabolism such as chlorophyll synthesis or degradation, lipid metabolism or vitamin synthesis.

### Identification of functional clusters within SDR families

For multigenic SDR families, the analyses can be conducted further with phylogenetic calculations. To illustrate the importance of this complementary approach, we focused on two large families involved in secondary metabolism: SDR110C (ABA2 xanthoxin dehydrogenase family) and SDR108E (4-DFR) family. For tropinone reductase (SDR65C) and menthone/salutaridine reductase (SDR114C) families, readers are referred respectively to Brock *et al.*[37] and Ziegler *et al.*[38] for complete phylogenetic analyses.

**Figure 5:**
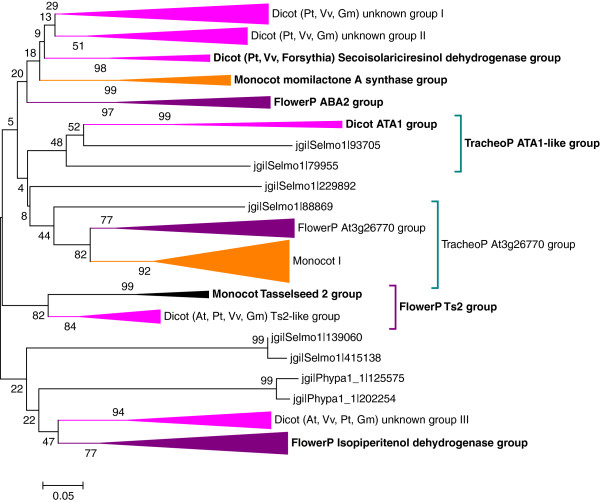
**Phylogenetic tree of the SDR110C family.** Amino acids sequences recognized by the SDR110C HMM were aligned with ClustalW algorithm. The evolutionary history and the bootstrap test (500 replicates) were computed as described for SDR108E (Figure 3). Full references of sequences compressed in different clusters are provided as supplemental data (Additional file 3: Figure S1B).

In our previous review [8], we listed six different functions described for SDR110C in the scientific literature: Arabidopsis ABA2 xanthoxin dehydrogenase (abscissic acid biosynthesis) [39,40], rice diterpenoid momilactone synthase A [41], mint (*Mintha sativa*) isopiperitenol dehydrogenase [42], *Forsythia intermedia* secoisolariciresinol dehydrogenase (lignan biosynthesis), the maize or rice feminization gene *TASSELSEED2*[43] and the Arabidopsis *AtATA1* gene, involved in pollen and anther tapetal cells development [44]. A phylogenetic tree was established from the analysed genome sequences and completed with mint isopiperitenol dehydrogenase and forsythia secoisolariciresinol dehydrogenase sequences (Figure 5 and Additional file 3: Figure S1B). Remarkably, the six functions described in the literature were distributed on five different clades, thus giving valuable hypotheses regarding the putative function of the orthologues or paralogues in other Angiosperm species. On the different clades, we also observe that highly homologous SDRs are often clustered in specific chromosomal regions, illustrating the importance of gene duplication events in the diversification process. At last, the different accessions of Selaginella are distributed in three distinct clades. However, the bootstrap values of the different nodes were too low to clearly establish a clear relationship between lycophytes and Angiosperms SDR110C sequences.

For the highly variable SDR108E family, we included the SDR115E family in the analysis as both family are closely related (see above). As reported for the SDR110C family, several branches can be associated with functions described in the literature [8]: 4-DFR [33], anthocyanidin reductase (AnR) [34,35], HC-toxin reductase [31], phenylacetaldehyde reductase [45], cinnamoyl-CoA reductase (CCR) [46] or eutypine reductase [47] (Figure 3). In contrast to SDR110C, the tree is also informative concerning the evolution of SDRs among land plant since distinct sequences from *S. moellendorffii* and *P. patens* are clearly associated with independent clades. These associations are of special interest for certain classes of enzymes such as the CCR catalysing the first irreversible oxidation step leading to monolignol synthesis. Indeed, several enzymes involved in the lignin biosynthesis pathway appeared early in land plant evolution and the moss are believed to accumulate uncondensed monolignols [48]. Thus, the association on the same branch of sequences from *P. patens* and *S. moellendorffii* with Angiosperms *bona fide* CCR suggests that the enzyme anciently acquired its specificity and diverged rapidly from other SDR108E members. Last, as observed for SDR110C, several highly similar genes are clustered in specific chromosomal regions. Hence, with numerous members and a low conservation rate of amino acid sequences, the SDR108E family and its daughter branch SDR115E constitute a good example of a gradual and fast evolution of a multigenic family. Since the majority of the described enzymes reduce phenolic compounds, we may hypothesize that the SDR108E evolution accompanied tightly the complexification of phenolic and phenylpropanoid metabolism during land plant radiation.

**Figure 6:**
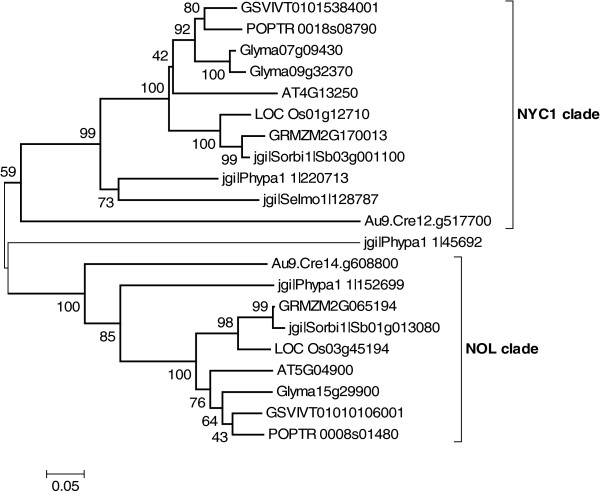
**Phylogenetic trees of the chlorophyllase b (NYC1/NOL) family.** Amino acids sequences from the AT4G13250 family were aligned with ClustalW algorithm. The evolutionary history and the bootstrap test (500 replicates) were computed as described for SDR108E (Figure 3).

Although essential for the functional study of large SDR families, phylogenetic analyses may be very informative for smaller families as well. This is the case of the Non-Yellow-Coloring 1 (NYC1) chlorophyllase b family, where the phylogenetic analyses clearly divide the family in two distinct clades: NYC1 and NOL (Non-yellow-coloring-Like) that diverged from a common ancestor (Figure 6). It was suggested that during evolution, the divergence led to the emergence of a functional hetero-oligomer, since both genes are necessary for chlorophyll b degradation [49,50].

**Figure 7:**
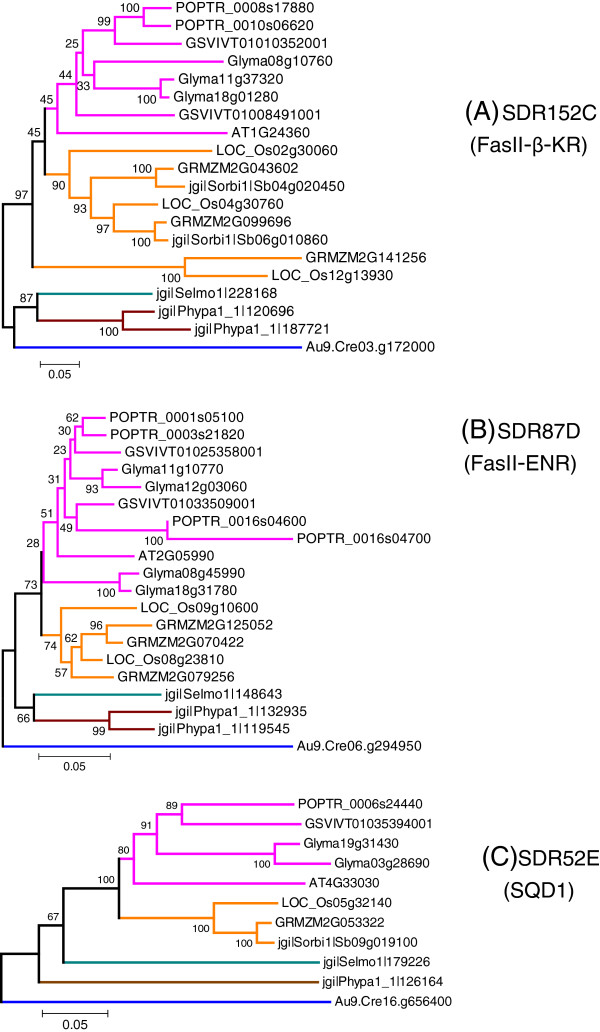
**Phylogenetic trees of three families involved in lipid primary metabolism.** (**A**) SDR152C-FasII-β-keto-reductase (β-KR); (**B**): SDR87D-FasII-Enoyl-ACP-reductase (ENR); (**C**) SDR52E UDP-sulfoquinovose synthase (SQD1). Amino acids sequences recognized by the SDR152C, SDR87D and SDR52E HMMs were aligned with ClustalW algorithm. The evolutionary history and the bootstrap test (500 replicates) were computed as described for SDR108E (Figure 3).

At last, we carried analyses on three SDR families involved in lipids primary metabolism: fatty-acid synthase (FAS-II)-β-ketoacyl synthase (SDR152C) [51], (FAS-II)-enoyl-ACP reductase (SDR87D) [52] and the UDP-sulfoquinovose synthase (SDR52E) involved in sulfolipids biosynthesis [53]. By contrast with the families involved in secondary metabolism discussed above, the average sequence identity is high (Table 3), ranging from 68% to 75%. When the N-terminal chloroplast peptide signals are removed from sequence alignments, the average identities reach the scores of 79% (SDR152C) and 84% (SDR87D and SDR 52E). Phylogenetic trees were deduced from sequence alignments (Figure 7). Despite the presence of some duplication events observed for SDR152C (Figure 7A) and SDR87D (Figure 7B), the tree topologies are in good agreement with plant taxonomy for all the three SDR families, thus suggesting that the primary structure has been conserved under a high pressure of selection.

## Conclusions

Work presented in this paper aimed at providing a full picture of plant SDRs using the current classification, especially the recent SDR nomenclature initiative. The combination of HMM models and similarity searches enabled us to classify most of the plant SDRs into a core of 49 families. Of these 49 families, 42 could be associated to an HMM, while the other 7 families being only defined on the basis of amino acids sequence conservation. Remarkably, all predicted SDRs from Angiosperms or *S. Moellendorffii* (corresponding to the so-called ‘higher plants’) could be categorized within these families. As all families exhibit a high degree of primary structure conservation, the average amino acid identities ranging from 37% to 87% among plant genomes, all SDRs sequences from Angiosperms can be analysed easily on the sole basis of sequence alignment, using very classical software (Blast, Multialin, ClustalW). For moss *P. patens* and green alga *C. reinhardtii* sequences, the predictions are less accurate, 3% and 20% of predicted SDRs remain unclassified. This limitation probably results from the under-representation of bryophyte and chlorophyte sequences compared to Angiosperms. In addition, the development of genome sequencing on more distant taxa (for example charophytes, liverworts or hornworts) should increase the number of UNIPROT sequences with sufficient divergences, thus improving the quality of HMM and allowing, in a mid-term, the definition of HMMs for the orphan SDR families.

Strikingly, the number of families found in Angiosperms (49) does not differ much from the 47 SDR families listed in the human genome [5]. The large proportion of families (35 out of 49) found in all Viridiplantae, from Algae to Angiosperms, is consistent with the view that most SDR sub-branches diverged early during evolution [54]. Plants possess either SDRs common to all Eukaryotes or SDRs of bacterial origin, in particular SDRs deriving from the plastidial endosymbiosis. However, the major difference between plants and other eukaryotes, that explains the high number of SDRs in ‘higher plants’, lies in the existence of large multigenic families. These families expanded much later during evolution, as attested by their under-representation in moss and algae. Because of their involvement in secondary metabolism routes (including hormone biosynthesis), they can be considered as an adaptative character that emerged during land colonization and emergence of the vascular apparatus.

## Abbreviations

AnR: Anthocyanidin reductase; A. thaliana: Arabidopsis thaliana; C. reinhardtii: Chlamydomonas reinhardtii; 4-DFR: Dihydroflavonol 4-reductase; G. max: Glycine max; HMM: Hidden Markov model; IFR: Isoflavone reductase; O. sativa: Oryza sativa; P. patens: Physcomitrella patens; P. trichocarpa: Populus trichocarpa; PLR: Pinoresinol reductase; S. bicolor: Sorghum bicolor; S. moellendorffii: Selaginella moellendorffii; SDR: Short-chain dehydrogenase/reductase; V. vinifera: Vitis vinifera; Z. mays: Zea mays.

## Competing interests

The authors declare that they have no competing interests.

## Author’ contributions

HM participated in sequence alignments, phylogenetic analyses and collection of functional annotations. LB initiated the SDR inventory and participated in sequence alignments. BVDR conceived the study, participated in its design and coordination and drafted the manuscript. YK and BP led the HMM analyses and helped to draft the manuscript. All authors read and approved the final manuscript.

## Supplementary Material

Additional file 1 **Table S1.** Exhaustive inventory of plant SDRs. Click here for file

Additional file 2 **Table S2.** List of ambiguous predictions (proteins only recognized by a single HMM with a low score). Click here for file

Additional file 3 **Figure S1.** Full phylogenetic trees of SDR108E (A) and SDR110C (B) families. The evolutionary history was inferred using the Neighbor-Joining method. The percentage of replicate trees in which the associated taxa clustered together in the bootstrap test (500 replicates) are shown next to the branches. Click here for file
