# Supplementary figures and images for: The Plant Short-Chain Dehydrogenase (SDR) superfamily: genome-wide inventory and diversification patterns
Source: BMC Plant Biol. 2012 Nov 20;12:219. doi: 10.1186/1471-2229-12-219 (PMC3541173; doi:10.1186/1471-2229-12-219)

## Slide 1
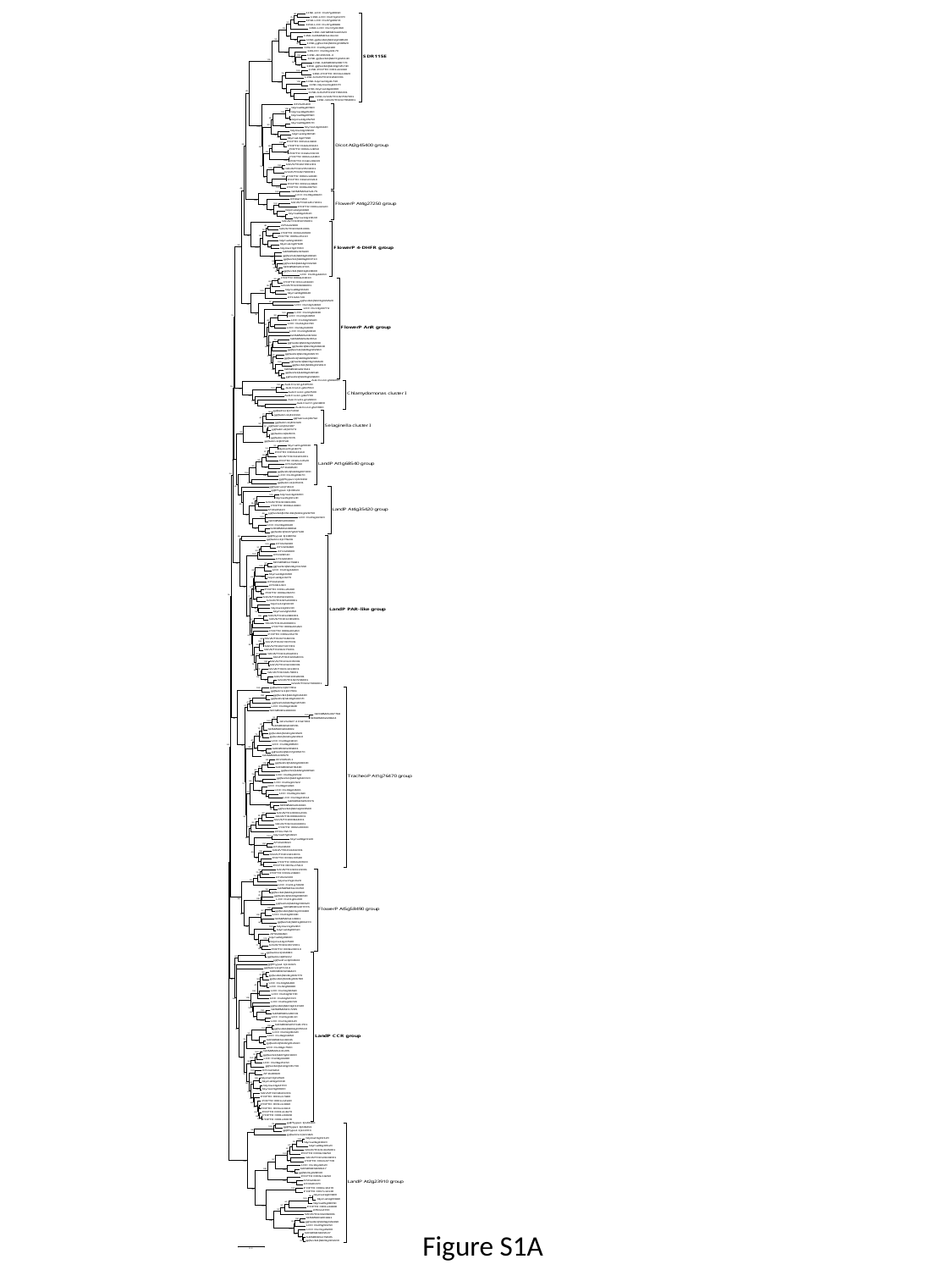

Figure S1A

## Slide 2
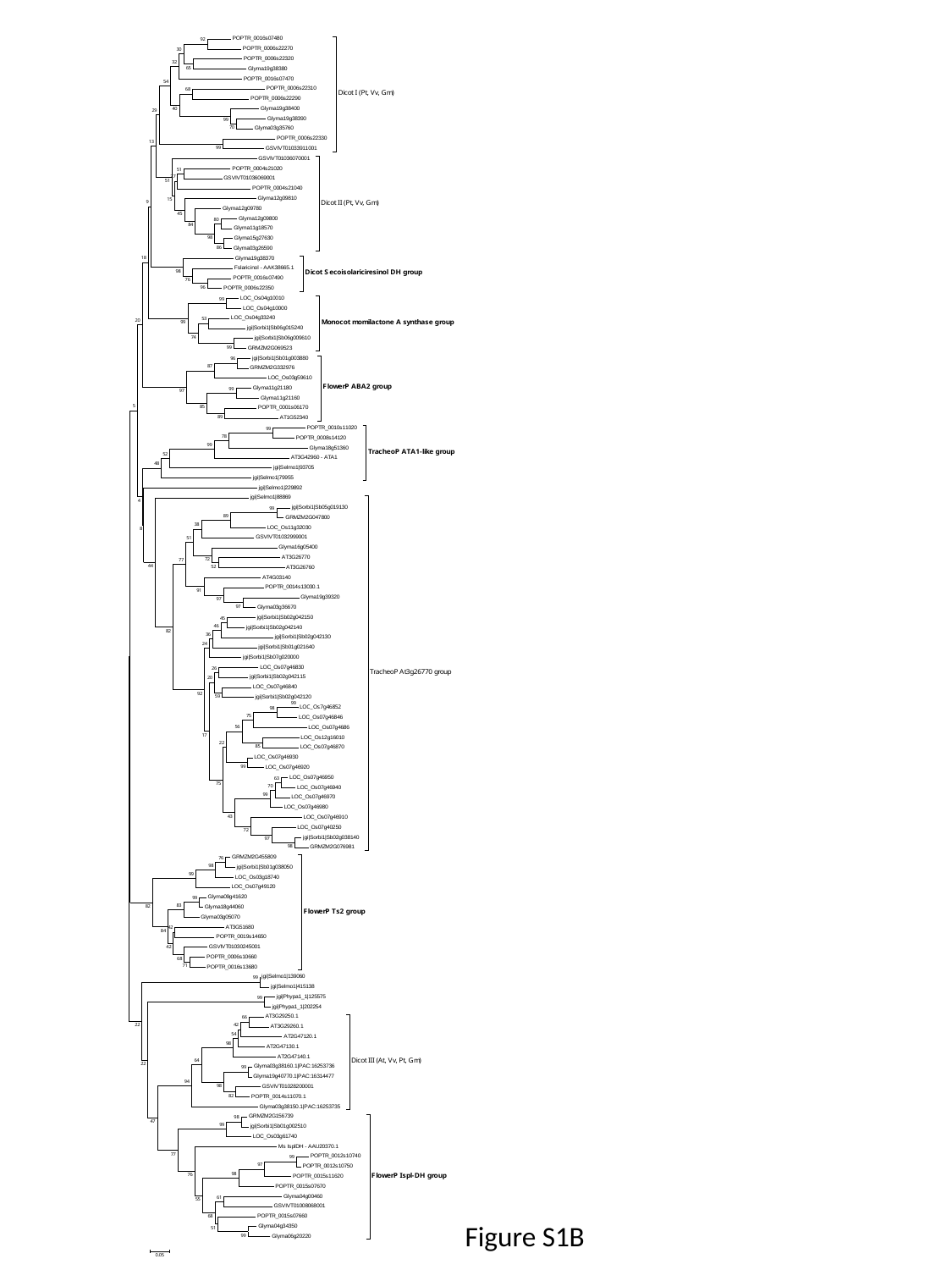

Figure S1B

Supplement: Additional file 3 — Figure S1. Full phylogenetic trees of SDR108E (A) and SDR110C (B) families. The evolutionary history was inferred using the Neighbor-Joining method. The percentage of replicate trees in which the associated taxa clustered together in the bootstrap test (500 replicates) are shown next to the branches. [file 1471-2229-12-219-S3.ppt]
